# How does the antimicrobial stewardship provider role affect prospective audit and feedback acceptance for restricted antibiotics in a Canadian tertiary-care center?

**DOI:** 10.1017/ice.2023.152

**Published:** 2024-02

**Authors:** Keely M. Hammond, Dima Kabbani, Karen E. Doucette, Stephanie W. Smith, Cecilia Lau, Serena Bains, Karen Fong, Jackson Stewart, Justin Z. Chen

**Affiliations:** 1 Core Internal Medicine Program, Department of Medicine, Faculty of Medicine and Dentistry, University of Alberta, Edmonton, Alberta, Canada; 2 Division of Infectious Diseases, Department of Medicine, Faculty of Medicine and Dentistry, University of Alberta, Edmonton, Alberta, Canada; 3 Pharmacy Services, Alberta Health Services, Edmonton, Alberta, Canada

## Abstract

Of 731 restricted antimicrobial prescriptions subject to antimicrobial stewardship program (ASP) prospective audit and feedback (PAF) over a 3-year period, 598 PAF recommendations (82%) were fully accepted. Physician auditors had an increased odds of PAF recommendation acceptance, reinforcing the complementary role of the ASP physician in the multidisciplinary ASP team.

Prospective audit and feedback (PAF) interventions often include a multidisciplinary team of pharmacists, physicians, and trainees.^1^ Rates of acceptance of PAF recommendations can vary; the influence of specific provider roles on PAF acceptance rates has not been extensively studied.^1,2^

A PAF intervention was implemented in 2018 at the University of Alberta Hospital, a 700-bed, tertiary-care center in Edmonton, Canada. Prescriptions of 6 restricted antimicrobials (meropenem, imipenem, ertapenem, daptomycin, linezolid, and tigecycline) were eligible for audit. Prescriptions for surgical prophylaxis were excluded. PAF was performed on weekdays by a team of antimicrobial stewardship program (ASP) pharmacists (an equivalent 4-year Entry-to-Practice Bachelor of Science Pharmacy degree and a 1-year postgraduate hospital pharmacy accredited residency program), ASP physicians [Royal College of Physicians and Surgeons of Canada certified in infectious diseases (ID)] and supervised postgraduate ID or medical microbiology physician trainees during clinical ASP rotations.

Real-time written and verbal feedback was provided to the primary team via 2 methods: a chart note in the legal record of care plus 1 of 3 secondary forms of communication [ie, in person, by telephone, or via electronic medical record using Epic Secure Chat (Epic, Verona, WI)] to a member of the primary team able to execute real-time clinical decisions.

The modality of the secondary form of communication was chosen by the auditor; more than one modality could be used until the primary team was reached. If the audit was performed by a physician-pharmacist team, either or both providers may have provided feedback. Feedback provided by a trainee was supervised directly by the auditing physician. Otherwise, ASP providers performing the audit alone provided feedback alone. In this study, we investigated the effect of audit provider type on PAF recommendation acceptance.

## Methods

We performed a retrospective review of all prescriptions subject to PAF between April 2018 and March 2021. PAF recommendations were categorized by audit provider type and acceptance outcomes. Acceptance was determined at 24 hours after the recommendation.

The primary outcome was the percentage of prescriptions with fully accepted PAF recommendations among the total number of prescriptions with actionable ASP recommendations. The secondary outcome was the odds ratio (OR) of acceptance between 4 ASP provider types: physician–pharmacist team, physician alone, pharmacist alone, and supervised trainee.

The Pearson χ^2^ test was performed on multiple predictors with respect to recommendation acceptance: auditing provider type (pharmacist–physician team, physician, pharmacist, and physician trainee), auditor gender, type of recommendation (stop, change, or other), prescribing service (medicine, critical care, or surgery), ID service involvement, and antimicrobial audited (carbapenem or noncarbapenem). Using logistic regression, we determined the odds ratios (ORs) of recommendation acceptance for each PAF provider role. The same 6 variables listed above were included in the multivariable model, producing an adjusted OR (aOR). Variables were examined for significant interactions using the Woolf test. Statistical analyses were performed using R Studio software (2021, R Foundation for Statistical Computing, Vienna, Austria).

The University of Alberta Research Ethics Board granted ethics approval (no. Pro00110892). The study was not funded.

## Results

Of 1,896 total prescriptions audited during the 3-year study period, 731 (39%) had actionable PAF recommendations. Carbapenems accounted for 677 prescriptions (93%), predominantly meropenem prescriptions (n = 545, 75%). Moreover 437 prescriptions (60%) originated in medicine programs, 197 (27%) originated in surgical care, and 97 (13%) originated in critical care. In total, 372 prescriptions (51%) were written by physician trainees, 263 (36%) were written by staff physicians, and 89 (12%) were written by other prescriber roles. The ID service was involved in the patient’s care in 137 prescriptions (19%). Table 1 includes the proportion of audits performed by each auditor type and sex.

**Table 1.** Acceptance of Prospective Audit and Feedback Recommendations by Acceptance Predictor

Variable	Recommendation Acceptance			*P* Value
	No, No. (%)	Yes, No. (%)	Total	
Total	133 (18)	598 (82)	731	
**PAF provider type**				.088
ASP pharmacist–physician team^a^	57 (21)	211 (79)	268	
ASP physician (n=4)^b^	19 (12)	141 (88)	160	
ASP pharmacist (n=4)^c^	37 (18)	171 (82)	208	
Physician trainee (n=12)^d^	20 (21)	75 (79)	95	
**PAF provider sex**				.392
Female (n=12)	76	307	383	
Male (n=8)	32	177	209	
Mixed (team)	25	114	139	
**Type of recommendation**				<.001^f^
Change^e^	85 (19)	452 (81)	537	
Discontinue	41 (32)	87 (68)	128	
Other	7 (11)	59 (89)	66	
**Prescribing service**				.303
Medicine	82 (17)	399 (83)	481	
Critical care	17 (17)	82 (83)	99	
Surgery	34 (23)	117 (77)	151	
**ID involvement**				.171
No	102 (17)	492 (83)	594	
Yes	31 (22)	106 (78)	137	
**Antimicrobial**				1.000
Carbapenem	123 (18)	554 (82)	677	
Noncarbapenem	10 (19)	44 (81)	54	

Note. Values are reported as absolute number of audits and percentage within the category in parentheses. The *P* value for the Pearson χ^2^ statistic is reported for each variable.

a Physician–pharmacist team auditors recommended “discontinue” in 52 instances (19%).

b Physician auditors recommended “discontinue” in 31 instances (19%).

c Pharmacist auditors recommended “discontinue” in 26 instances (13%).

d Physician trainee auditors recommended “discontinue” in 19 instances (20%).

e We examined a subset of “change” recommendations to assess whether the intensity of the change recommendation (ie, the difference in spectrum of the proposed alternative antimicrobial from the original) was associated with recommendation acceptance. To do this, we examined all recommendations to change empiric (excluding culture-directed) therapy (n = 352). Within this subset, there was no significant difference in recommendation acceptance between recommendations to change to another broad-spectrum choice (most commonly piperacillin-tazobactam) or change the dose only (n = 291) and recommendations to change to a narrower-spectrum agent than piperacillin-tazobactam (n = 61) (82% vs 79% acceptance; *P* = .607).

f *P* value <.05.

Recommendations were fully accepted in 598 instances (82%), partially accepted in 30 instances (4%), and not accepted in 103 instances (14%). A “discontinue” recommendation was accepted less often than a “change” or “other” (Table 1). For each auditor type, there were no differences in the proportions of “discontinue” recommendations made (*P* = .140) (Table 1). In the logistic regression analysis, physician auditors were associated with a significant OR in favor of acceptance compared to pharmacist-physician teams (OR, 2.00; 95% CI, 1.16–3.59; *P* = .015; aOR, 2.09; 95% CI, 1.14–3.93; *P* = .020) (Table 2). PAF recommendations to discontinue an antimicrobial were associated with decreased odds of acceptance when compared with a change recommendation (OR, 0.40; 95% CI, 0.26–0.62; *P* < .001; aOR, 0.39; 95% CI, 0.25–0.62; *P* < .001).

**Table 2.** Unadjusted Odds Ratios (OR) and Adjusted Odds Ratios (aOR) of Prospective Audit and Feedback Recommendation Acceptance Predictor

Variable	OR	95% CI	*P* Value	aOR	95% CI	*P* Value
**PAF provider type**						
RV = Pharmacist–physician team	1			1		
Physician^a^	2.00	1.16–3.59	.015	2.09	1.14–3.93	.020
Pharmacist	1.25	0.79–1.99	.345	1.18	0.71–1.97	.536
Physician trainee	1.01	0.58–1.83	.965	1.04	0.59–1.91	.885
**PAF provider gender**						
Female	1			1		
Male	1.34	0.88–2.18	.173	1.12	0.70–1.86	.626
Mixed (team)	1.13	0.69–1.89	.635	1.27	0.75–2.21	.378
**Type of recommendation**						
RV = Change	1			1		
Discontinue^a^	0.40	0.26–0.62	<.001	0.39	0.25–0.62	<.001
Other	1.59	0.75–3.91	.269	1.56	0.72–3.88	.293
**Prescribing service**						
RV = Medicine	1			1		
Critical care	0.99	0.57–1.81	.976	0.87	0.49–1.61	.647
Surgery	0.71	0.45–1.12	.131	0.73	0.47–1.17	.193
**ID involvement**						
RV = ID not involved	1			1		
ID involved	0.71	0.45–1.13	.137	0.65	0.41–1.05	.074
**Antimicrobial**						
RV = Noncarbapenem	1			1		
Carbapenem	1.02	0.48–2.10	.949	0.76	0.34–1.56	.482

Note. CI, confidence interval; RV, reference value; ID, infectious diseases.

a Significant 95% confidence interval and *P* value < .05.

The reason for declining PAF recommendations was documented in only 121 prescriptions (17%); the most common reason was attending team preference (80%). Other reasons included waiting for ID consult (11%), palliation (4%), patient transfers (3%), and dose changes (2%).

A subgroup analysis of 463 ASP providers performing audits alone was also completed assuming the same provider delivered the PAF recommendation. Because this assumption would be unreliable in a pharmacist–physician team, in which feedback could have been delivered by either a provider type alone or simultaneously as a team, a regression model excluding the pharmacist–physician team was performed. Physician auditors were associated with slightly increased odds of acceptance compared to pharmacists in the adjusted but not the unadjusted analysis (OR, 1.61; 95% CI, 0.89–2.97; *P* = .120; aOR, 1.85; 95% CI, 1.00–3.51; *P* = .053).

## Discussion

The overall PAF recommendation acceptance rate in our cohort was 82%, which was relatively high.^3,4^ Various strategies can improve acceptance, including the use of in-person communication of recommendations and motivational interviewing.^3,5–7^ We hypothesized that our center’s high acceptance rate was influenced by the strategy of always delivering PAF recommendations via 2 forms of communication. The modality of using 2 forms of communication was based on the ability to successfully reach a primary team member able to execute clinical decisions.

The recommendation acceptance rate remained high among critical care (n = 84, 87%) and medicine prescribers (n = 347, 82%), and the acceptance rate among surgical services was only slightly lower (n = 157, 80%), similar to other Canadian institutions.^2^ ID service involvement did not affect the odds of acceptance. Our findings align with those of other studies noting decreased odds of acceptance with recommendations to discontinue compared to changing the agent or regimen.^2^ Overall, the heterogeneity in acceptance rates likely reflects differences in prescribing culture and environmental factors at each institution.

Auditor roles were individually evaluated and show mixed results in terms of acceptance.^8–10^ High PAF acceptance and overall program success have been previously reported with pharmacist-delivered PAF programs; thus, the role of the multidisciplinary team cannot be overstated.^10^ We did not find any prior studies examining acceptance of PAF provided by supervised physician trainees; however, acceptance rates decreased when PAF was performed by unsupervised ID physician trainees.^9^ Our findings highlight the importance of the multidisciplinary ASP team but reinforce the complementary role of the physician in PAF.

Our study had several limitations, including the retrospective study design. The absence of randomization in the modality of secondary communication limited our analysis of its influence on PAF recommendation acceptance. This factor will be the basis of future study at our institution. Also, we did not comprehensively examine all patient-related factors (ie, illness severity), prescriber-related factors (ie, years of experience), or PAF-related factors (ie, focus on diagnosis versus antimicrobial regimen optimization, or number of simultaneous recommendations), and these factors may have influenced acceptance.

In summary, PAF recommendation acceptance rates were high in our cohort. Our study highlights the importance of a multidisciplinary ASP team in PAF but reinforces the complementary role of the ASP physician. Further studies are required to better understand factors that influence PAF acceptance.
